# Breastfeeding Progression in Preterm Infants Is Influenced by Factors in Infants, Mothers and Clinical Practice: The Results of a National Cohort Study with High Breastfeeding Initiation Rates

**DOI:** 10.1371/journal.pone.0108208

**Published:** 2014-09-24

**Authors:** Ragnhild Maastrup, Bo Moelholm Hansen, Hanne Kronborg, Susanne Norby Bojesen, Karin Hallum, Annemi Frandsen, Anne Kyhnaeb, Inge Svarer, Inger Hallström

**Affiliations:** 1 Knowledge Centre for Breastfeeding Infants with Special Needs at Department of Neonatology, Copenhagen University Hospital Rigshospitalet, Copenhagen, Denmark; 2 Department of Health Sciences, Faculty of Medicine, Lund University, Lund, Sweden; 3 Danish National Panel of Experts on Breastfeeding Infants with Special Needs, Copenhagen, Denmark; 4 Department of Neonatology, Copenhagen University Hospital Herlev, Herlev, Denmark; 5 Department of Public Health, Section of Nursing, University of Aarhus, Aarhus, Denmark; 6 Department of Neonatology, Viborg Regional Hospital, Viborg, Denmark; 7 Paediatric Department, Holbaek University Hospital, Holbaek, Denmark; 8 Department of Neonatology, Copenhagen University Hospital Hvidovre, Hvidovre, Denmark; 9 Department of Neonatology, Odense University Hospital, Odense, Denmark; Canadian Agency for Drugs and Technologies in Health, Canada

## Abstract

**Background and Aim:**

Many preterm infants are not capable of exclusive breastfeeding from birth. To guide mothers in breastfeeding, it is important to know when preterm infants can initiate breastfeeding and progress. The aim was to analyse postmenstrual age (PMA) at breastfeeding milestones in different preterm gestational age (GA) groups, to describe rates of breastfeeding duration at pre-defined times, as well as analyse factors associated with PMA at the establishment of exclusive breastfeeding.

**Methods:**

The study was part of a prospective survey of a national Danish cohort of preterm infants based on questionnaires and structured telephone interviews, including 1,221 mothers and their 1,488 preterm infants with GA of 24–36 weeks.

**Results:**

Of the preterm infants, 99% initiated breastfeeding and 68% were discharged exclusively breastfed. Breastfeeding milestones were generally reached at different PMAs for different GA groups, but preterm infants were able to initiate breastfeeding at early times, with some delay in infants less than GA 32 weeks. Very preterm infants had lowest mean PMA (35.5 weeks) at first complete breastfeed, and moderate preterm infants had lowest mean PMA at the establishment of exclusive breastfeeding (36.4 weeks). Admitting mothers to the NICU together with the infant and minimising the use of a pacifier during breastfeeding transition were associated with 1.6 (95% CI 0.4–2.8) and 1.2 days (95% CI 0.1–2.3) earlier establishment of exclusive breastfeeding respectively. Infants that were small for gestational age were associated with 5.6 days (95% CI 4.1–7.0) later establishment of exclusive breastfeeding.

**Conclusion:**

Breastfeeding competence is not developed at a fixed PMA, but is influenced by multiple factors in infants, mothers and clinical practice. Admitting mothers together with their infants to the NICU and minimising the use of pacifiers may contribute to earlier establishment of exclusive breastfeeding.

## Background

Breastfeeding is important to preterm infants, as it seems to provide even more pronouced health benefits than to infants born at term age [1], [2], [3], [4], [5], [6]. Even though there are significant variations in preterm breastfeeding rates between countries [7], [8], [9], [10], [11], [12], [13], [14] and neonatal intensive care units (NICUs) within coutries [7], [14] preterm infants are not breastfed to the same extent as term infants [7]. Thus in many countries the breastfeeding process is not initiated to the same extent as in term infants [8], [9], [10], [11], [15], [16], [17], [18], and even though the mother tries to establish breastfeeding, the success rates measured as breastfeeding at discharge are relatively lower [12], [13], [14]. Furthermore, studies find that the duration of breastfeeding of preterm infants is also shorter compared to infants born at term [13], [19], [20], and one study found that infants born at the lowest gestational age (GA) were breastfed for the shortest duration [13]. Some of the benefits of breast milk in very preterm infants during the first weeks of life are well recognised, such as reduced risk of NEC [2], [21] and infections [1], which is why breastfeeding guidance is an important part of neonatal nursing care [22], [23].

To promote the breastfeeding process in preterm infants, knowledge of the expected postmenstrual age (PMA) at which the majority of preterm infants reach different breastfeeding milestones, together with an individual assessment, is an important tool for supporting mothers in breastfeeding their infants.

### Breastfeeding milestones

Feeding milestones in breastfed preterm infants, defined as the age at which a skill is achieved [26], have not been well-studied compared to bottle-fed preterm infants [23], [24], [25], [26], [27], [28], and since infant sucking skills seems to differ between bottle-and breastfeeding, data gathered from bottle-feeding are not necessarily applicable to breastfeeding [29].

Feeding milestones for preterm infants are commonly described as initiation of oral feeding/breastfeeding and the achievement of full oral feeding/exclusive breastfeeding [12], [23], [24], [27], [28], [30]. However other milestones might be considered. Skin-to-skin contact is the first step in the breastfeeding process, as it promotes breastfeeding behaviour [31] and is positively associated with breastfeeding duration in preterm infants [32]. Another milestone is the nutritive sucking of a fixed number of mls [12], [23], [24], [25], [28], which requires test-weighing in breastfed infants, something that is not used routinely at all NICUs [22].

### Postmenstrual age

Most preterm infants are not able to breastfeed exclusively at birth because of immaturity and/or illness. A Swedish study of preterm infants with GA 26–35 weeks found that breastfeeding in clinically stable preterm singleton infants was initiated from 27.9 weeks PMA, and exclusive breastfeeding was established at median 36.0 weeks PMA [24]; infants with GA 26–31 weeks established exclusive breastfeeding slightly earlier (35.7 weeks) [23]. The studies were, however, based on small numbers of infants, making it difficult to determine whether PMA at the establishment of exclusive breastfeeding differed significantly according to GA; also, the studies included only preterm singletons without severe morbidity. Thus research is lacking about what can be expected in breastfeeding progress for infants with low and high GA, including both singletons and multiples. It has not been investigated whether preterm infants reach the various breastfeeding milestones at a fixed PMA regardless of GA or whether clinical procedures and other factors are associated with the PMA at establishment of exclusive breastfeeding.

## Aims

The aims were to describe rates of breastfeeding duration at pre-defined times in various preterm gestational age groups, to analyse the postmenstrual age at breastfeeding milestones in various preterm GA groups, and to analyse factors associated with postmenstrual age at establishment of exclusive breastfeeding.

## Methods

### Ethics Statement

The study was conducted in accordance with the Declaration of Helsinki [33] and approved by the Danish Data Protection Agency (j.nr. 2009-41-4024); surveys do not, according to Danish law, need to be approved by the Biomedical Research Ethics Committee. Mothers gave their written informed consent to participate.

### Design

The study was part of a prospective survey of a national Danish cohort of preterm infants based on questionnaires and structured telephone interviews conducted from September 2009 to December 2011. This article is the second from the cohort; the first article analysed factors associated with exclusive breastfeeding at discharge and adequate duration [14].

### Setting

Denmark, with its 5.5 million inhabitants, has about 60,000 births per year, 7% of which are premature. Denmark has public health care, and all citizens enjoy easy and equal access to health care in public hospitals free of charge [34].

Most of the preterm infants are admitted to one of Denmark's 19 NICUs, except for stable late preterm infants born after 35+0 gestational weeks, who are cared for in postpartum wards. Four NICUs provide high-intensive care, 14 medium-intensive care, and one low-intensive care [22].

The breastfeeding support given at Danish NICUs includes skin-to-skin contact, support of breast milk expression, rooming-in for at least a part of the infant's hospitalisation and support of the parents' presence [22]. Most Danish NICUs use a transition strategy with scheduled feedings and decreased tube feedings but different assessment methods (test-weighing or estimate by the mother/nurse). Preterm infants are hospitalised until breastfeeding is well established or exclusive breastfeeding is given up and mixed feeding or bottle-feeding is established [22].

### Instruments

Based on a review of the literature and a national expert panel, three study-specific questionnaires for mothers of preterm infants were developed to explore rates and progress of breastfeeding in preterm infants and the use of various clinical practises to facilitate breastfeeding. The national expert panel consisted of eight neonatal nurses with experience in the breastfeeding of preterm infants and research, four of whom were International Board Certified Lactation Consultants.

The questionnaires included background questions and questions about the infant's breastfeeding progression, the mother's breastfeeding experience and clinical practice. Questionnaire 1 with 38 questions was answered by mothers approximately one week after delivery, and questionnaire 2 with 59 questions was answered by the mother at the infant's discharge from NICU to home. Questionnaire 3 with 17 questions was used for structured telephone interviews with breastfeeding mothers at the infant's 1, 4, 6 and 12 months corrected age or until breastfeeding ceased, whichever occurred first. More details and the questionnaires can be found in a previously published paper [14].

### Participants and data collection

Inclusion criteria were preterm infants of less than 37 gestational weeks who were admitted to a NICU during the first five days of life from 1 September 2009 to 31 August 2010, as well as their mothers. Exclusion criteria were infant discharge to maternity units before five days of age, interpreter not available for a non-Danish speaking mother or neonatal death. Mothers who did not plan to breastfeed and in addition did not initiate breast milk expression participated only with the first questionnaire, and their data were not included in the analyses in the present paper.

All departments in Denmark that routinely take care of preterm infants during breastfeeding establishment participated in the study, which included 18 of the 19 NICUs, two special care units and one children's department; 18 of the 21 participating units adhered to the project protocol [14]. The data set used in the present paper is publicly available at the Danish Data Archive on request for researchers who meet the criteria for access to confidential data.

### Outcomes

Outcomes selected for the present study were:

- The percentage of infants initiating skin-to-skin contact during five different time periods.
- PMA at breastfeeding milestones as described below. As not all Danish NICUs use test-weighing routinely, the PMA at first complete breastfeeding was selected as a breastfeeding milestone between initiation of breastfeeding and exclusive breastfeeding for the present study.
- The percentage of infants initiating breastfeeding and exclusively breastfed at 1, 4 and 6 months, as well as performing any breastfeeding at 1, 4, 6 and 12 months of chronological and corrected age.

### Definitions of terminology

Breastfeeding

- Exclusive breastfeeding was defined as an infant feeding directly at and from the breast, and can include medication and vitamins.
- Any breastfeeding included other feeding methods (such as bottle, cup, lact-aid, regardless of content) in addition to directly breastfeeding.
- For telephone follow-up the infants were regarded exclusively breastfed when they were only feeding at and from the breast, besides breastfeeding water and/or a maximum of one formula feed could be given a week.

Breastfeeding milestones were defined as the PMA at

- Breastfeeding initiation, defined as the mother's description of when the baby was first placed at the breast for licking, tasting and maybe latching, but not necessarily sucking and sinking.
- First complete breastfeeding, defined as the mother's description of when the baby first completed breastfeeding (the prescribed volume, or if it was deemed that the infant did not need supplementation feeding). For infants not exclusively breastfed, this milestone was the “first complete oral feeding”.
- Establishment of exclusive breastfeeding defined as the mother's description of when the baby took all at and from the breast. For infants not exclusively breastfed, this milestone was “full oral feeding”.
- Discharge from NICU to home.

Skin-to-skin contact was defined as the infant – wearing only a diaper and maybe a cap and socks – lying on its parent's bare chest.

Preterm infant gestational age groups (GA groups) were divided into four groups depending on gestational age (GA) in weeks + days [35], [36]:

- Extremely preterm infants: GA 24+0 – 27+6.
- Very preterm infants: GA 28+0 – 31+6.
- Moderate preterm infants: GA 32+0 – 34+6.
- Late preterm infants: GA 35+0 – 36+6.

For this study, the lower limit for late preterm infants was set at 35+0 weeks and days, given that preterm infants with GA less than 35+0 weeks and days are admitted routinely to NICUs in Denmark regardless of the their physical situation.

Age definitions [37]:

- Chronological age  =  postnatal age (PNA): time elapsed from birth.
- Postmenstrual age (PMA): gestational age plus chronological age.
- Corrected age: chronological age reduced by the number of weeks born before 40 weeks of gestation.

### Statistical analyses

SPSS version 21.0 was used for statistical analyses. Descriptive statistics were used to describe mother and infant characteristics. The normally distributed results are reported with mean and standard deviation (SD); the remaining results are reported with median, interquartile range (IQR) or percentages [38]. One-way ANOVA was used to determine statistically significant differences in normally distributed scale data between GA groups. Pearson's Chi-Square test was used to determine statistically significant differences for nominal data. A scatter plot was performed and a curve was fitted for correlation between GA and PMA at the establishment of exclusive breastfeeding. Breastfeeding duration for infants lost to follow up were adjusted to the time at the latest answered questionnaire and the analyses performed with all infants.

PMA at the establishment of exclusive breastfeeding was analysed by linear regression models. The explanatory variables were first analysed in univariate models; subsequently the variables with a p-value of less than 0.2 were analysed simultaneously in a multiple stepwise backward model stepwise removing variables with p<0.05. The regression analyses were performed with one infant per mother to ensure that mothers of twins were not counted twice [38]; for multiple births, the first born infant was included. Values of p<0.05 were considered statistically significant.

The explanatory variables included in the stepwise backward general linear regression model were

- GA in weeks, broken down into four groups as descibed above
- Multiple births
- Being small for gestational age (SGA), defined as birth weight more than two standard deviations (SD) smaller than expected according to GA
- Mechanical ventilation
- First-time mothers
- Mother speaking another language than Scandinavian at home
- Admitting the mother together with the infant in the NICU directly after delivery
- Skin-to-skin contact on a daily basis after incubator care
- Pacifier use during hospitalisation, broken down into three groups: no use of a pacifier, minimising the use of a pacifier during breastfeeding transition, and unrestricted use of a pacifier
- One or more of the following practices may be regarded as minimising the use of a pacifier during the transition from tube-feeding to breastfeeding: predominantly using the pacifier during tube-feedings, painful or stressful events, predominantly using the pacifier in the mother's absence, or removing the pacifier completely

## Results

### Participant selection

**Figure 1:**
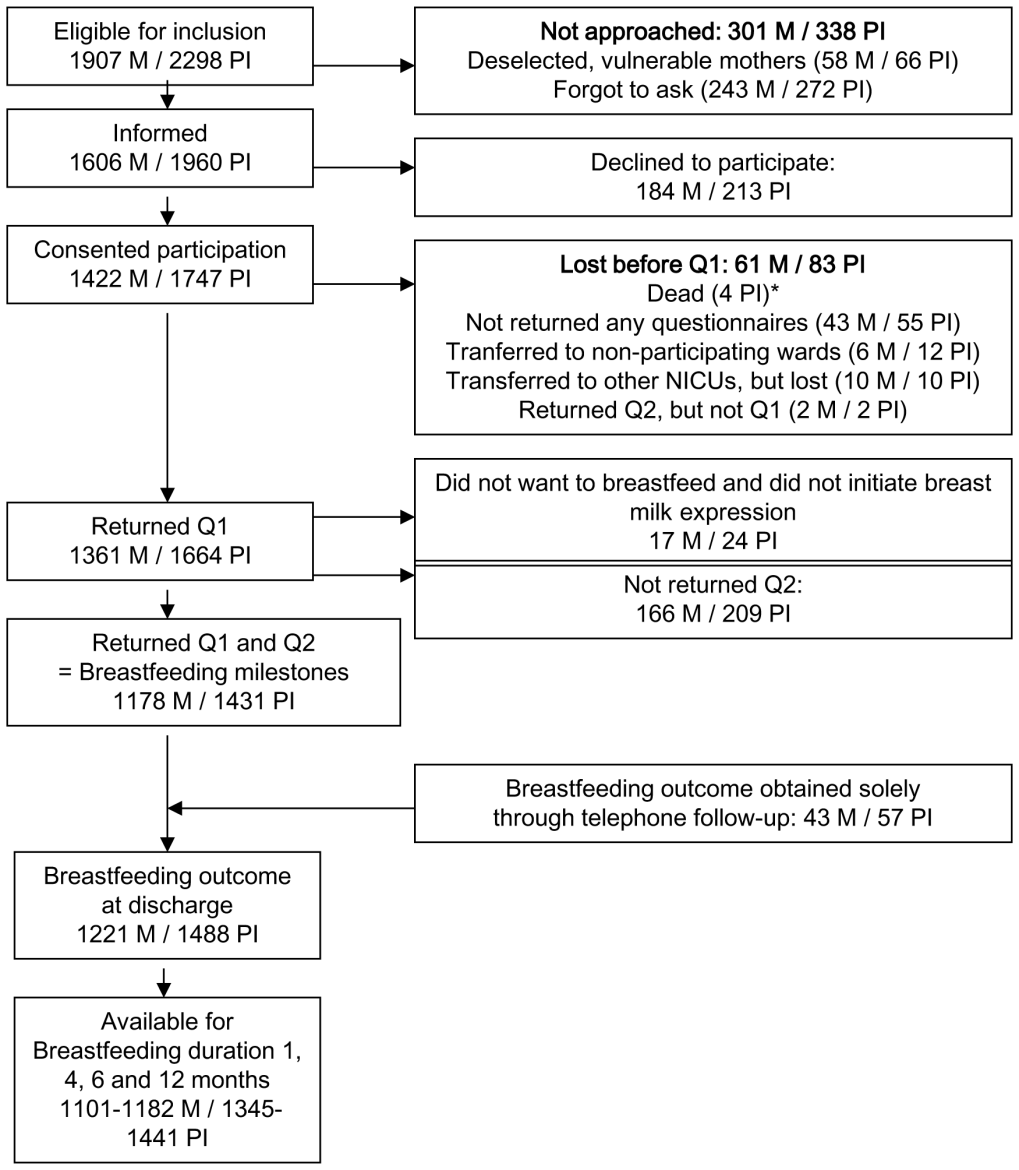
Flowchart. BF  =  breastfeeding, M  =  mothers, NICU  =  Neonatal Intensive Care Unit, PI  =  preterm infants, Q  =  Questionnaire. *The four infants who died after inclusion were all twins; no mothers were lost due to infant death.

Selection of participants is described in the flow chart (see Figure 1). Data on breastfeeding at discharge were available for 1,488 infants (65% of those eligible) and 1,221 mothers. Data on breastfeeding duration at 1, 4, 6 and 12 months were available for between 1,345 and 1,441 infants at the various times (90–97% of the 1,488 infants).

Significantly fewer of mothers of extremely preterm infants eligible for inclusion participated at discharge from NICU compared to mothers of older infants (p<0.0001). Of mothers participating with questionnaire 1, significantly more of those who did not return questionnaire 2 had a lower level of education (p<0.001).

Table 1 shows mother and infant characteristics. Mothers had a mean age of 31; nearly all mothers had planned to breastfeed. Of the infants, 60 were extremely preterm, 257 were very preterm, 688 were moderate preterm, and 483 were late preterm; 36% were multiples. When the mothers had planned to breastfeed and/or initiated breast milk expression, 99% of the infants initiated breastfeeding. Twenty-one per cent of the infants had their first breastfeeding experience in combination with CPAP treatment, with significantly more of the extremely preterm infants (62%) suckling with CPAP (p<0.0001). In Denmark, the use of mechanical ventilation is minimised by early nasal CPAP and INSURE (INtubate SURfactant Extubate) [39], thus, only 6% of infants were mechanically ventilated.

**Table 1:** Infant and maternal characteristics.

		Total	GA 24–27	GA 28–31	GA 32–34	GA 35–36	
Cohort data N		population	n = 60	n = 257	n = 688	n = 483	p-value
**Infant**							
Median gestational age (IQR), weeks + days	1488	34+1	26+5	30+4	33+6	36+6	
		(32+2–35+2)	(25+4–27+3)	(29+3–31+2)	(33+0–34+3)	(35+2–36+3)	
Multiple birth, %	1488	36	33	32	37	36	
SGA, %	1474	18	17	21	15	21	
Gender, boys, %	1488	51	55	55	53	47	
Initiated breastfeeding, %	1471	99	95	99	99	100	<0.0001
Mechanical ventilation, %	1401	6	60	11	3	3	<0.0001
Nasal CPAP treatment, %	1387	59	100	97	60	33	<0.0001
Minimising the use of a pacifier during BF transition, %	1386	28	26	33	31	22	0.001
**Mother**							
Mean age, years (SD)	1219	31 (5)	31 (5)	31 (5)	31 (5)	31 (5)	
Lives together with infant's father, %	1219	96	98	94	96	96	
Danish/Scandinavian origin, %	1215	93	92	93	93	93	
Education, high (>16 years), %	1207	20	20	21	20	19	
Education, intermediate (14–16 years), %		47	56	46	46	48	
Education, low or none (<14 years), %		33	24	34	34	34	
Smoking, %	1210	10	12	9	10	11	
Primiparous, %	1171	65	70	72	63	63	
Mode of delivery, caesarean section, %	1219	50	53	60	49	47	0.014
Mother admitted into the NICU directly from birth, %	1207	29	4	15	26	42	<0.0001
Planned to breastfeed, %	1213	99	96	99	99	99	
Spouse supports breastfeeding plans, %	1210	97	100	95	97	98	
Breastfed other infants excl>4 months, %	1171	17	19	17	16	17	

Excl  =  exclusively, NICU  =  Neonatal Intensive Care Unit, N  =  Number included in analyses, n  =  subgroup numbers, SD  =  standard deviation, SGA  =  small for gestational age

### Age at breastfeeding milestones

Of all the infants 27% initiated skin-to-skin contact immediately postpartum (pp), 27% did so later but within 6 hours pp, 27% did so later than six hours pp but during the first 24 hours of life (Table 2), with significantly fewer of extremely and very preterm infants initiating skin-to-skin contact within 24 hours of birth (p<0.0001).

**Table 2:** Breastfeeding milestones for various gestational age groups.

		Total								
	N	population	n	GA 24–27	n	GA 28–31	n	GA 32–34	n	GA 35–36
Skin-to-skin contact immediately after delivery	1481	27%	1	2%	18	7%	204	30%	176	37%
from minutes to 6 hours pp		27%	2	3%	44	17%	220	32%	136	29%
6–24 hours pp		27%	11	18%	101	40%	188	27%	100	21%
24–48 hours pp		10%	6	10%	60	23%	48	7%	27	6%
More than 48 hours pp		7%	31	52%	33	13%	15	2%	21	4%
Not skin-to-skin with mother within 7 days		3%	9	15%	0	0%	12	2%	18	4%
		*Mean (SD)*		*Mean (SD)*		*Mean (SD)*		*Mean (SD)*		*Mean (SD)*
Birth weight (grams)	1330	2094 (582)	47	833 (158)	211	1443 (301)	617	2128 (412)	455	2480 (454)
Initiation of breastfeeding, PMA (weeks)	1344	34.4 (1.8)	48	31.8 (2.3)	213	32.0 (1.3)	619	34.2 (1.0)	464	36.1 (0.7)
Initiation of breastfeeding, weight (grams)	1300	2113 (530)	45	1329 (452)	203	1527 (294)	599	2103 (409)	453	2466 (435)
First complete breastfeeding, PMA (weeks)	1047	36.1 (1.2)	35	36.3 (2.1)	170	35.5 (1.5)	507	35.8 (1.0)	335	37.0 (0.8)
Establishment of exclusive breastfeeding, PMA (weeks)	1002	36.7 (1.2)	29	37.5 (2.0)	162	36.6 (1.6)	479	36.3 (1.1)	332	37.3 (0.9)
Discharged exclusively breastfed, PMA (weeks)	974	37.5 (1.6)	29	39.8 (3.0)	149	37.6 (1.8)	462	37.1 (1.5)	334	37.7 (0.9)
Discharge weight exclusively breastfed (grams)	913	2583 (374)	28	2655 (489)	141	2580 (376)	438	2568 (368)	306	2600 (371)
Initiation of bottle-feeding, PMA (weeks)	460	36.8 (1.6)		37.4 (2.4)		36.1 (2.0)		36.6 (1.5)		37.4 (1.0)
		*Median*		*Median*		*Median*		*Median*		*Median*
		*(IQR)*		*(IQR)*		*(IQR)*		*(IQR)*		*(IQR)*
Initiation of breastfeeding, PNA (days)	1344	1 (0–5)		39 (21–51)		8 (4–17)		2 (1–4)		0 (0–1)
First complete breastfeeding, PNA (days)	1047	13 (8–22)		66 (56–78)		34 (26–45)		14 (10–19)		7 (4–11)
Establishment of exclusive breastfeeding, PNA (days)	1002	16 (11–25)		78 (64–89)		41 (32–52)		17 (13–22)		10 (6–13)
Discharge exclusively breastfed, PNA (days)	974	19 (13–31)		86 (73–102)		48 (37–61)		20 (16–27)		12 (8–16)

GA  =  gestational age, IQR, interquartile range, PMA  =  postmenstrual age, PNA  =  Postnatal age, pp  =  postpartum

**Figure 2:**
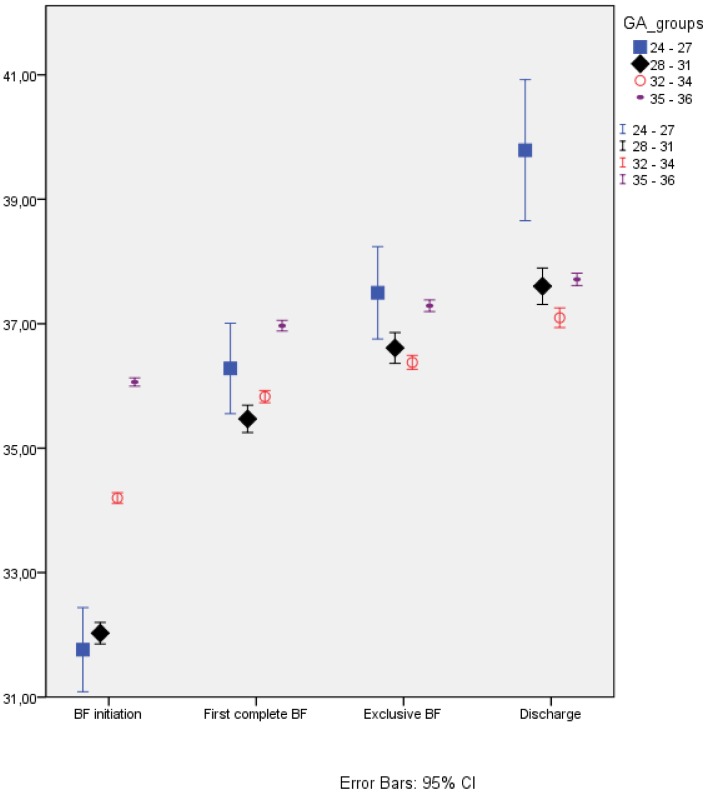
Breastfeeding milestones for various gestational groups. BF  = breastfeeding, CI  =  confidence interval, GA groups  =  gestational age groups.

Extremely and very preterm infants initiated breastfeeding at mean PMA 31.8 and 32.0 weeks respectively (Figure 2). Of the extremely preterm infants, 21% (n = 10) initiated breastfeeding before PMA 30 weeks. The median postnatal age (PNA) was inversevely related to GA as the extremely, very, moderate and late preterm infants were 39, eight, two and zero postnatal days respectively at breastfeeding initiation (p<0.0001) (Table 2).

**Figure 3:**
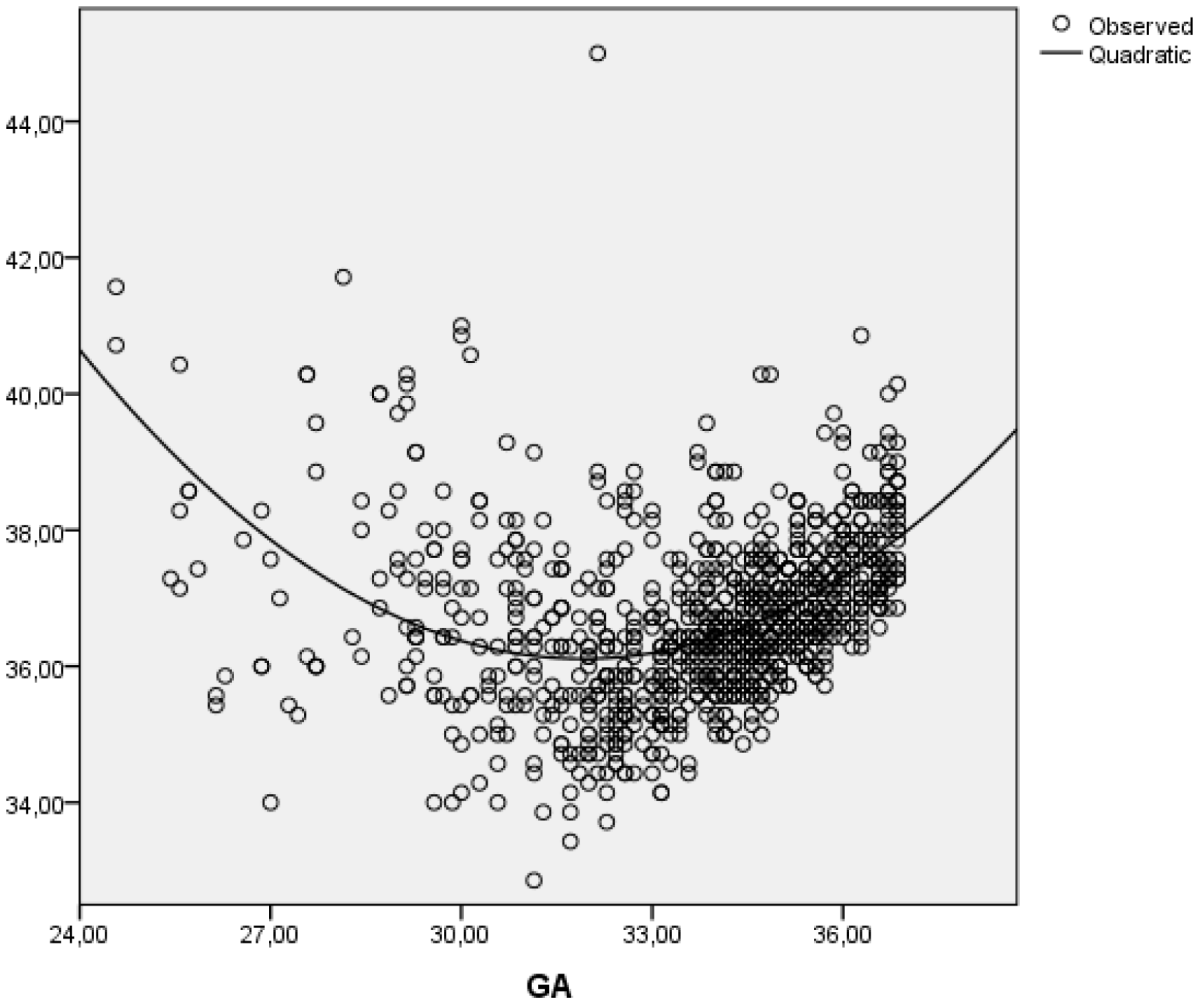
Postmenstrual age at establishment of exclusive breastfeeding compared to gestational age (GA) at birth.

The very preterm infants reached the milestone “first complete breastfeed” at the significantly lowest mean PMA (35.5 weeks), whereas the moderate preterm infants had the significantly lowest mean PMA (36.3 weeks) at the establishment of breastfeeding (Table 2). The best fit describing the correlation between GA and PMA at the establishment of exclusive breastfeeding was a quadratic curve (Figure 3), showing that infants with low and high GAs established breastfeeding at higher PMA than infants in between. Of the infants who established exclusive breastfeeding, 98% did so before corrected term age, with 32.9 weeks as the lowest observed PMA.

The extremely preterm infants had the longest transition time from breastfeeding initiation to discharge and the largest variation in PMA at each breastfeeding milestone. The late preterm infants had the most accelerated breastfeeding progress, as shown in Figure 2.

Infants in all GA groups had significantly different mean PMA at all breastfeeding milestones, except the extremely preterm infants, whose mean PMA was not significantly different from the mean PMA of the very preterm infants at breastfeeding initiation, neither from the moderate preterm infants at first complete breastfeeding nor from the late preterm infants at establishment of exclusive breastfeeding (Figure 2). At establishment of exclusive breastfeeding, the very and moderate preterm infants did not differ in mean PMA. At discharge, the very preterm and late preterm infants did not differ in mean PMA. All GA groups had significantly different median PNAs at each breastfeeding milestone.

### Factors associated with PMA at establishment of exclusive breastfeeding

**Figure 4:**
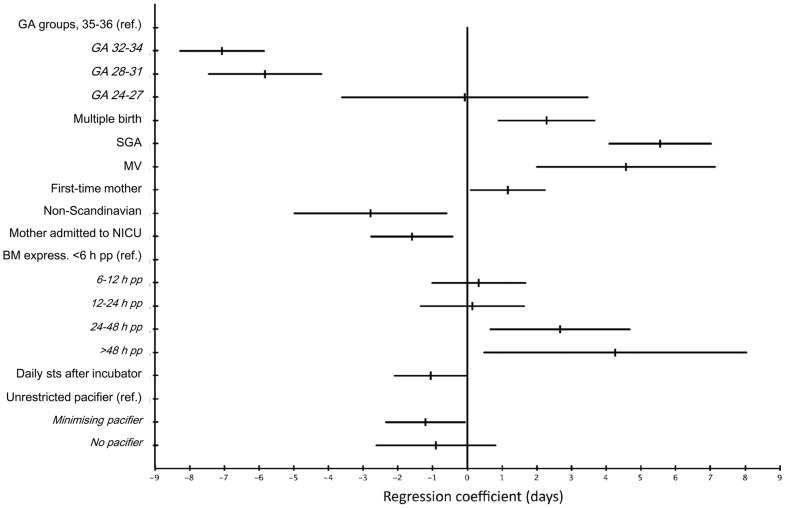
Adjusted model of factors associated with PMA at establishment of exclusive breastfeeding. BM  =  breast milk, GA  =  gestational age, MV  =  mechanical ventilation, NICU  =  neonatal intensive care unit, SGA  =  small for gestational age, sts  =  skin-to-skin contact

A multiple linear regression analysis with 749 of the 858 mother-infant pairs where the PMA was known, showed that factors significantly associated with later establishment of exclusive breastfeeding were small for gestational age (SGA) (5.6 days (95% CI 4.1–7.0)), multiple birth (2.3 days (95% CI 0.9–3.7)), infant having been mechanically ventilated (4.6 days (95% CI 2.0–7.1)), first-time mother (1.2 days later (95% CI 0.1–2.2)), and initiating breast milk expression later than 24 hours after delivery (see Table 3). Factors significantly associated with earlier establishment of exclusive breastfeeding were mother speaking another language than Scandinivian in her home (2.8 days (95% CI 0.6–5.0)), admitting a mother to the NICU together with her infant directly after delivery (1.6 days (95% CI 0.4–2.8)), minimising the use of a pacifier during breastfeeding transition (1.2 days (95% CI 0.1–2.3)), and continuing skin-to-skin contact on a daily basis after incubator care (1.1 days (95% CI 0.0–2.1)) (see Figure 4). The model could explain 29% of the difference in PMA at the establishment of exclusive breastfeeding.

**Table 3:** Linear regression of factors associated with PMA at establishment of exclusive breastfeeding.

			Unadjusted analyses		Adjusted analysis	
One infant per mother	N	Prev. %	Days (95% CI)	p-value	Days (95% CI)	p-value
**Later establishment of exclusive breastfeeding, days**						
Multiple birth	858	17	2.5 (1.1–4.0)	0.0008	2.3 (0.9–3.7)	0.001
Small for gestational age	851	14	6.5 (5.0–8.1)	<0.0001	5.6 (4.1–7.0)	<0.0001
Boys	858	51	0.1 (−1.1 to 1.2)	0.945		
Mechanical ventilation	843	5	5.2 (2.7–8.7)	0.0001	4.6 (2.0–7.1)	0.0005
First time mothers	824	64	1.3 (0.1–2.4)	0.036	1.2 (0.1–2.2)	0.03
Mode of delivery, caesarean section	857	47	1.2 (0.1–2.4)	0.031		
Nipple shield use	844	51	0.2 (−0.9 to 1.3)	0.743		
Test weighing at most breastfeeds	850	32	0.2 (−1.0 to 1.4)	0.704		
First breast milk expression, *<6 hours pp (ref)*	830	23	0		0	
* 6–12 hours pp*		41	1.2 (−0.3–2.7)	0.11	0.3 (−1.0–1.7)	0.63
* 12–24 hours pp*		24	1.3 (−0.4–2.9)	0.13	0.1 (−1.3–1.6)	0.85
* 24–48 hours pp*		10	5.3 (3.1–7.4)	<0.0001	2.7 (0.7–4.7)	0.009
*>48 hours pp*		2	7.1 (3.0–11.2)	0.0008	4.3 (0.5–8.0)	0.03
**Earlier establishment of exclusive breastfeeding, days**						
Gestational age groups, *GA 24*–*27 weeks*	858	3	−3.1 (−6.4–0.1)	0.06	0.1 (−3.5–3.6)	0.97
* GA 28–31 weeks*		16	5.3 (3.7–6.8)	<0.0001	5.8 (4.2–7.4)	<0.0001
* GA 32–34 weeks*		48	6.9 (5.8–8.1)	<0.0001	7.1 (5.9–8.3)	<0.0001
* GA 35–36 weeks (ref)*		33	0		0	
Education, *high (ref)*	850	21	0			
*Intermediate*		47	0.5 (−1.0 to 2.0)	0.499		
*Low*		32	0.9 (−0.7 to 2.5)	0.274		
Maternal smoking	852	7	0.3 (−1.6 to 2.3)	0.750		
Mother admitted together with infant to the NICU	851	30	1.0 (−0.2–2.5)	0.10	1.6 (0.4–2.8)	0.007
Skin-to-skin contact on a daily basis after incubator care	851	57	1.5 (0.3–2.6)	0.011	1.1 (0.0–2.1)	0.046
Pacifier use, *no pacifier*	839	13	0.9 (−0.9–2.6)	0.331	0.9 (−0.8–2.6)	0.30
*Minimising the use of a pacifier during BF establishment*		33	2.1 (0.8–3.3)	0.001	1.2 (0.1–2.3)	0.04
*Unrestricted use of a pacifier (ref)*		54	0		0	
Mother speaking another language than Scandinavian at home	854	7	2.2 (0.0–4.5)	0.047	2.8 (0.6–5.0)	0.01

BF  =  breastfeeding, GA  =  gestational age, pp  =  postpartum

### Feeding methods at breastfeeding milestones

**Figure 5:**
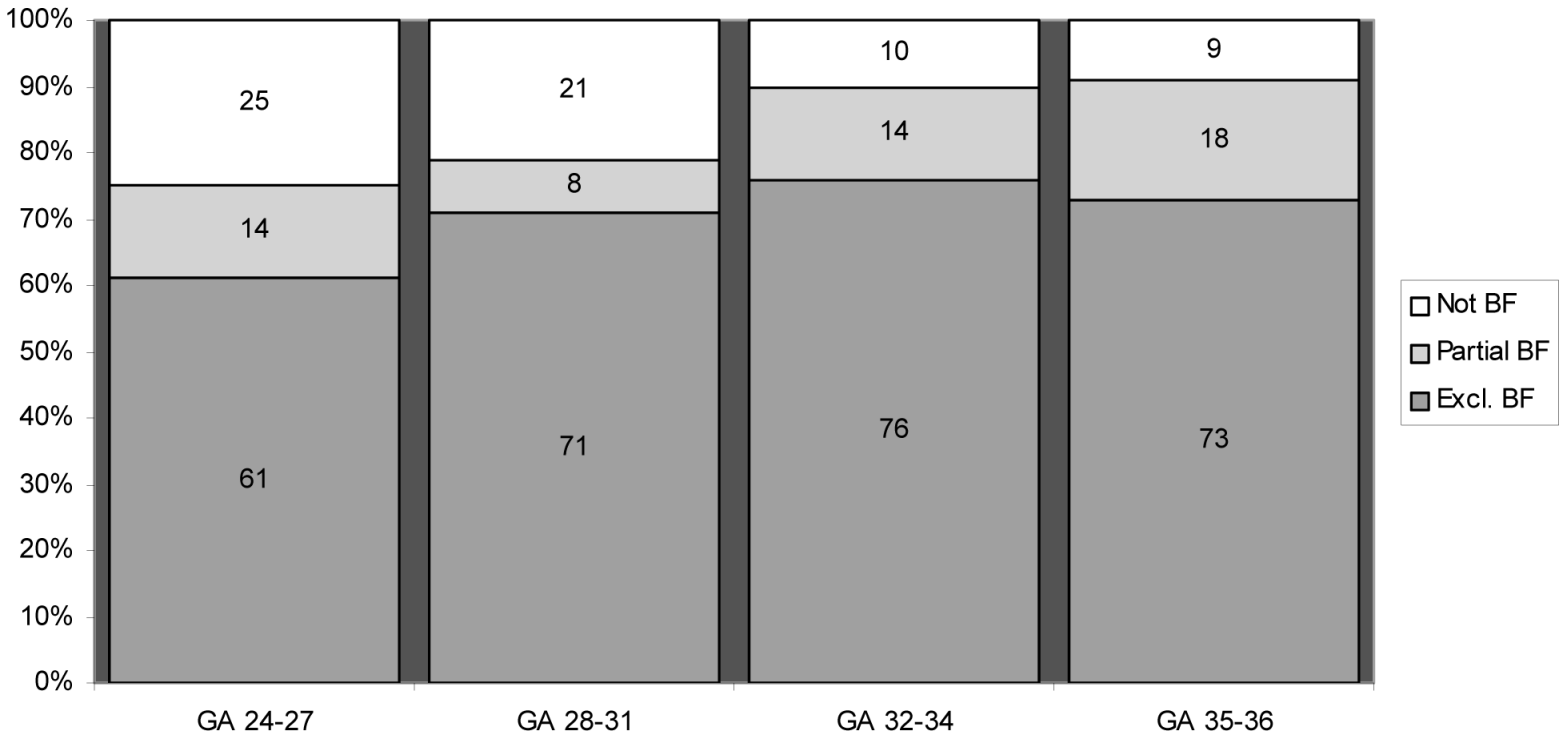
Feeding method at full oral feeding for various gestational age groups. BF  =  Breastfeeding, excl  =  exclusive, GA  =  gestational age

At the first complete oral feeding 79% of the infants were breastfed, 8% fed from a combination of breast and bottle/cup and 13% were only bottle-fed. Of the infants solely breastfed at the first complete feeding, fewer than 2% were fed by bottle at any feeding session the rest of that day and the majority (66%) were fed by a combination of breast and tube feeding. At the milestone of full oral feeding, 73% of the infants were exclusively breastfed, with significantly fewer of extremely preterm infants being exclusively breastfed (p<0.0001) (see Figure 5). At discharge, 68% of the infants were exclusively breastfed and 17% were partially breastfed. Again, significantly fewer (50%) of the extremely preterm infants were exclusively breastfed (p<0.001). Of the infants exclusively breastfed at discharge, significantly more (95%) had their first complete feeding solely from the breast (p<0.0001).

### Breastfeeding duration at 1, 4, 6 and 12 months of age

For the total infant population, 13% were exclusively breastfed at six months chronological age, and including partially breastfed infants, 44% were breastfed at this time (Table 4). At six months corrected age, the corresponding percentages were 2% and 34%. At six months corrected age significantly more of the infants who were exclusively breastfed at discharge were still breastfeeding to any extent compared to infants who were exclusively breast milk fed (some or all of the breast milk in bottle) at discharge (45 and 23% respectively) (p<0.0001).

**Table 4:** Breastfeeding duration for various gestational age groups.

	N	Total	GA 24–27	GA 28–31	GA 32–34	GA 35–36	
	1488	population	n = 60	n = 257	n = 688	n = 483	
**Exclusive BF PNA**							
Exclusive BF PNA 1 month	1488	66	63*	73*	65	64	
Exclusive BF PNA 4 months	1488	38	38	37	37	40	
Exclusive BF PNA 6 months	1488	13	27	23	11	8	p<0.0001
**Any BF PNA**							
Any BF PNA 1 month	1488	85	95*	89*	84	82	p = 0.01
Any BF PNA 4 months	1488	57	62	59	57	56	
Any BF PNA 6 months	1488	44	50	47	43	44	
Any BF PNA 12 months	1488	12	8	14	12	10	
**Exclusive BF corrected age**							
Exclusive BF 1 month corr. age	1488	46	35	41	46	51	p = 0.016
Exclusive BF 4 months corr. age	1488	19	12	17	16	24	p = 0.004
Exclusive BF 6 months corr. age	1488	2	0	2	2	2	
**Any BF corrected age**							
Any BF 1 month corr. age	1488	68	60	63	69	69	
Any BF 4 months corr. age	1488	47	40	46	47	50	
Any BF 6 months corr. age	1488	34	28	30	33	36	
Any BF 12 months corr. age	1488	5	3	7	5	4	

BF  =  breastfeeding, corr  =  corrected, PNA  =  postnatal age (chronological age).

'*If an infant initiated breastfeeding, the duration was calculated form birth, likewise if exclusive breastfeeding was established. Thus some of theese infants were tube-fed expressed breast milk at this time point. The percentage represents infants, whose mothers' had not given up exclusive/any breastfeeding before one month.

At a glance, more extremely preterm infants were breastfed at chronological age times and fewer at corrected age times and this pattern was significant for exclusive breastfeeding at one and four months corrected age, as well as six months chronological age (Table 4).

## Discussion

In the present study, almost all preterm infants of mothers who planned to breastfeed (99%) initiated breastfeeding at the NICU and 79% of the infants performed their first complete oral feeding at the breast. These rates are higher compared to the preterm initiation rates in the U.S. (62%) and Australia (80%–86%) [10], [11], [12] and similar to the initiation rate of term infants in Denmark (99%) [18]. The high initiation rates in the present study may be due to Danish NICUs' high priority of breastfeeding support, as reflected in their self reported practices of early skin-to-skin contact, breast milk expression, parental presence, restricted use of bottle-feeding [22] and the cultural norm of breastfeeding initiation [18]. When the first complete feeding was exclusively at the breast, bottle-feeding for the rest of the feedings that day was rare in the present study, with less than 2% of the infants fed by bottle. These results support the NICUs' self-reported restricted use of bottle-feeding [22] and may contribute to the relatively high rate of exclusively breastfed infants at discharge.

Skin-to-skin contact was widely used at the Danish NICUs and initiated by a large proportion (81%) of preterm infants during the first 24 hours of life, while an additional 16% initiated skin-to-skin contact later during the first week of life. Initiation time of skin-to-skin contact on a national level has been reported only in extremely preterm infants [40], showing a median initiation time of 6 days. In a smaller study of preterm infants with GA 28–34 weeks at two selected Swedish NICUs, 82% of the infants initiated skin-to-skin contact within the first 24 hours of life [41], which is comparable to the present study. The initiation time was not associated with PMA at establishment of exclusive breastfeeding.

The results of the breastfeeding milestones in our study show that it is possible for preterm infants to initiate breastfeeding at early times. Infants born before GA 32 weeks initiated breastfeeding at a mean PMA of approximately 32 weeks. However, breastfeeding progression was inversely related to GA; therefore, extremely and very preterm infants had higher PMA at establishment of exclusive breastfeeding. Still we believe that it is important to initiate breastfeeding at early times because it may promote breastfeeding at discharge [42]. The extremely and very preterm infants' higher PMA at establishment of exclusive breastfeeding may be related to slower or altered brain maturation caused by acute illness, nutrition, quality of experience or factors not yet known to science [43], [44], [45]. Thus the most preterm infants suffer from more morbidity, have longer stays in an incubator and longer hospitalisation [46].

In the present study, breastfeeding was initiated by 21% of the extremely preterm infants before PMA 30 weeks; early initiation has previously been reported in Sweden [23], [24] and is also in line with the “expansion of the Baby-Friendly Hospital Initiative to Neonatal intensive care”, which recommends that the only criterion for preterm breastfeeding initiation be infant stability, i.e., independent of birth weight, GA, PMA, and PNA [47]. The mean PMA at establishment of exclusive breastfeeding in the present study was 36.7 weeks overall, with extremely preterm infants having the highest mean PMA. The results are supported by a large Australian study in which preterm infants established exclusive suckle feeding at mean PMA 36.4 weeks and extremely preterm infants were found to have higher mean PMA [12]. Although the Australian study included infants who were both breastfed and bottle-fed, it found no significant differences in PMA between exclusively, partially and non-breastfed infants. A Swedish study showed earlier establishment of exclusive breastfeeding (median PMA 36.0 weeks) [24]; however, it lacked participation of preterm infants with servere mobidity, extremely preterm infants and preterm infants with gestational age of 36 weeks.

The present study shows that mean PMA and median PNA at different breastfeeding milestones differ according to GA group in a large national population of preterm infants. Breastfeeding competences are thus not developed at a fixed PMA nor a fixed PNA, but rather are influenced by multiple factors in infant, mother and clinical practice. We found that multiples, infants who were SGA, infants who had been mechanically ventilated, and infants of first-time mothers, when adjusted for GA groups, were delayed in PMA at exclusive breastfeeding establishment.

The clinical pratice of admitting mothers to the NICU together with the infant immediately after delivery was associated with earlier establishment of exclusive breastfeeding. The reason may be that the mother is able to observe and respond to the infant's early feeding cues, the opportunity for more breastfeeding sessions around the clock, and that mother and infant are not stressed out by separation, as rooming-in has shown to help parents feel as though they are a family and not just visitors to their own baby [48]. A Norwegian study found that when mothers were offered the chance to stay at the NICU for the infant's entire stay, more preterm infants were breastfed three months after discharge [49], and a Swedish study found that infants had significantly shorter hospital stays when parents were admitted to the NICU [50].

Minimisation of the use of a pacifier during breastfeeding transition was associated with earlier establishment of exclusive breastfeeding, which has not been researched before. It is reasonable to assume that infants who use a pacifier less at this stage are more keen to suck at the breast when offered and more likely to show hunger cues and breastfeed. The overall use of pacifiers has not been associated with the timing of full oral feeding [51]. Continued skin-to-skin contact on a daily basis after incubator care was also associated with earlier establishment of exclusive breastfeeding. It has been assumed that skin-to-skin contact supports development in preterm infants, as accelerated neurophysiological development has been reported in preterm infants receiving daily skin-to-skin contact [52]. We do not know the reasons why mothers who spoke another language than Scandinavian in their home established breastfeeding earlier, why this needs further investigation. Our results do not support the hypothesis that preterm infants need temporary facilitation of milk intake with use of a nipple shield [53], [54], as exclusive breastfeeding was established at a mean of PMA 36.7 weeks, and not earlier by infants using nipple shields. The difference in findings could be due to previous studies having been small (15 and 34 infants), with no comparison group, or measuring any breastfeeding instead of exclusive breastfeeding.

The signifcantly longer duration of exclusive breastfeeding at six months chronological age for extremely preterm infants has not been reported in other studies, and should be interpreted with caution because of the higher drop-out of these infants. The duration of breastfeeding at corrected age is comparable to other studies and exceeded by a Swedish study [13]. The six-month rate (13%) of exclusive breastfeeding for preterm infants in the present study is comparable to the 12% exclusive breast milk fed full-term Danish infants [55]. It is important that preterm infants establish exclusive breastfeeding at and from the breast at discharge as this affects breastfeeding duration.

The present study is, to our knowledge, the largest one that has been conducted of preterm breastfeeding milestones. These data might be able to help clinicians guide a mother in breastfeeding progression; however, we also think that the large variation in PMA within GA groups should encourge promotion of inidividualised assessment of the infant and inidividualised support for the mother.

### Strengths and limitations

The study is strengthened by its multicentre design, the large numbers of participants, the repeated telephone interviews to reduce re-call bias, and the reporting of direct breastfeeding, which often has lower rates than the rates of breast milk fed infants. Breastfeeding is often defined by WHO and many studies as breast milk feeding [9], [20], [56], [57], [58], [59].

A limitation is the high drop-out rate of extremely preterm infants. It is known that participants with poorer health outcomes are more reluctant to participate in surveys and drop out more often from cohorts [60]; that could indicate that even fewer of them established exclusive breastfeeding. The regression analysis was probably not affected by drop-out, as only exclusive breastfed infants were analysed, and the associations might persist. Another limitation is the weak definition of “first complete oral feeding”. To achieve more accurate data, a more narrow definition is needed. In a large national survey with infants at many different NICUs, several different feeding and transition strategies may be used. A more narrow definition may not give better data because it might be answered by fewer participants (not all infants were test-weighed). The weak definition of “first complete feeding” did not seem to influence the mothers, given that the variation in PMA at this milestone was not larger than for the other milestones, indicating that Danish mothers of preterm infants tend to interpret “first complete breastfeeding” in a similar way. Late preterm infants' higher PMA at establishment of exclusive breastfeeding could be due to the fact that most of those in the present study where admitted to a NICU because they needed neonatal care.

Breastfeeding rates in the present study may be biased both negatively and positively. We have reported the breastfeeding initiation rate of infants of mothers who planned to breastfeed and/or initiated breast milk expression. If infants of mothers who did not plan to breastfeed and did not initiate breastfeeding or breast milk expression were included in the analysis the breastfeeding initiation rate would decrease to 97%. The possibility that non-breastfeeding mothers declined to a greater degree to participate in the breastfeeding survey may have led to higher breastfeeding rates. This selection bias may also be present in other breastfeeding surveys with which we made comparisons [12], [13], [18]. The telephone interviews might have served as interventions in helping mothers breastfeed longer –although that was not the purpose of the interviews – given that they could ask questions of the NICU nurses conducting the interviews. Also, mothers could breastfeed longer because they were participating in a cohort study (known as the Hawthorne effect) [61]. On the other hand, exclusive breastfeeding duration may have been limited by being too pessimistic, considering that mothers of 117 infants, who where exclusively breastfed at discharge but completely weaned at first telephone interview, did not report duration of exclusive breastfeeding. Thus, duration was adjusted to the date of discharge.

## Conclusions

Danish mothers of preterm infants initiate breastfeeding to the same extent as mothers of term infants. Breastfeeding milestones are generally reached at different PNAs and PMAs depending on the GA group, but preterm infants are able to initiate breastfeeding within a few days depending on their physical condition. It might be expected that extremely preterm infants initiate breastfeeding at a higher PNA and, like SGA infants and infants of first-time mothers, establish exclusive breastfeeding at a higher PMA compared to other preterm infants, which is why patience is needed on the part of both mothers and staff. Admitting a mother directly to a NICU together with her infant and minimising the use of a pacifier during breastfeeding transition could contribute to earlier establishment of exclusive breastfeeding. So action should be taken to encourage these practices. Breastfeeding competences are not developed at a fixed PMA, but rather are influenced by multiple factors in the infant, mother and clinical practice. The present study indicates that if a mother wants to establish exclusive breastfeeding, bottle-feeding should not be introduced at the NICU.
